# Recombinant Human Sonic Hedgehog Protein Regulates the Expression of ZO-1 and Occludin by Activating Angiopoietin-1 in Stroke Damage

**DOI:** 10.1371/journal.pone.0068891

**Published:** 2013-07-23

**Authors:** Yuan-peng Xia, Quan-wei He, Ya-nan Li, Sheng-cai Chen, Ming Huang, Yong Wang, Yuan Gao, Yan Huang, Meng-die Wang, Ling Mao, Bo Hu

**Affiliations:** Department of Neurology, Union Hospital, Tongji Medical College, Huazhong University of Science and Technology, Wuhan, China; Rutgers University, United States of America

## Abstract

This study examines the regulating effect of Sonic Hedgehog (Shh) on the permeability of the blood-brain barrier (BBB) in cerebral ischemia. By employing permanent middle cerebral artery occlusion (pMCAO) model, we find that Shh significantly decreases brain edema and preserves BBB permeability. Moreover, Shh increases zonula occludens-1 (ZO-1), occludin and angiopiotetin-1 (Ang-1) expression in the ischemic penumbra. Blockage of Shh with cyclopamine abolishes the effects of Shh on brain edema, BBB permeability and ZO-1, occludin, Ang-1 expression. Primary brain microvessel endothelial cells (BMECs) and astrocytes were pre-treated with Shh, cyclopamine, Ang-1-neutralizing antibody, and subjected to oxygen-glucose deprivation (OGD). Results show that the Ang-1 protein level in the culture medium of Shh-treated astrocytes is significantly higher. Shh also increased ZO-1, occludin and Ang-1 expression in BMECs, while cyclopamine and Ang-1-neutralizing antibody inhibited the effects of Shh on the ZO-1 and occludin expression, respectively. This study suggests that, under ischemic insults, Shh triggers Ang-1 production predominantly in astrocytes, and the secreted Ang-1 acts on BMECs, thereby upregulating ZO-1 and occludin to repair the tight junction and ameliorate the brain edema and BBB leakage.

## Introduction

Stroke leads to the disruption of the blood-brain barrier (BBB), which increases the permeability of the brain microvasculature and eventually results in brain edema [1]. The principal structures that serve the function of the barrier are the tight junctions (TJs). TJs reduce the permeability of cerebral vessels by restricting the free molecular exchange between blood and brain tissues, and structural damage of TJs could cause the leakage of BBB and brain edema [2]. Zonula occludens-1 (ZO-1), occludin, claudin-5 proteins are important components of TJs structure and are implicated in the maintenance of integrity of TJs [3]. Therefore, understanding of the mechanism by which the integrity of TJs is maintained and the ZO-1, occludin, claudin-5 expression is regulated has potential implication for the treatment of cerebral ischemia.

A number of cytokines could mediate the change of BBB after cerebral ischemia. A recent study showed that the Sonic hedgehog (Shh), a glycoprotein secreted by astrocytes, interacts with cerebral endothelial cells to ensure the integrity of BBB by modulating the expression of ZO-1, occludin, claudin-5 [4]. Our previous studies exhibited that Shh is mainly secreted from astrocytes and could protect neurons against oxidative insults [5], [6]. Furthermore, Shh is transiently up-regulated in the focal ischemic brain [7], and inhibition of Shh signaling pathway aggravated brain edema in acute ischemic stroke [8]. But, the underlying mechanism by which Shh modulates the BBB to relieve brain edema in brain ischemia remains poorly understood.

Shh is functionally versatile during the vertebrate development. Shh signaling pathway is initiated when Shh binds with the specific receptor Patched-1, thereby releasing the transmembrane protein Smo and leading to activation of the transcription factor Gli-1, which induces the expression of downstream signaling pathway genes, including Patched-1 and Gli-1 [9]. Additionally, a Shh response element was identified in the NR2F2 promoter, which was different from Gli-1 [10]. Evidence obtained from the dental epithelia showed that ZO-1 may be the target of Gli-1 that controls cell size and polarity [11]. In the adult rat, Shh was also found to regulate the expression of many target genes involved in the development of blood vessel, such as angiopoietins [12].

Angiopoietins, including Angiopoietin-1, -2, -3, -4, play a major role in the development and integrity maintenance of blood vessels [13]. Angiopoietin-1 (Ang-1), which causes tightening of vessels by working on junctional molecules [14], is necessary for the stabilization and the maturation of growing blood vessels [15]. Furthermore, Ang-1 could substantially reduce endothelial permeability in vitro and ameliorate the BBB leakage in mice middle cerebral artery occlusion (MCAO) model [16], [17]. However, the molecular mechanism of Ang-1 on vascular permeability is still unknown.

A previous research showed that Shh up-regulates Ang-1 in fibroblasts [18]. Consistent with data that Shh induces Ang-1 in mesenchymal cells through activation of the orphan nuclear receptor, NR2F2 [10], our recent study demonstrated that Shh could upregulate the expression of Ang-1 in astrocytes under oxygen-glucose deprivation (OGD) by activating the NR2F2 [19]. On the basis of the findings, in this study, we examined the effects of Shh on the BBB integrity and the expression of Ang-1 and tight junction-associated proteins including ZO-1, occludin, claudin-5, in the rat model of permanent middle cerebral artery occlusion (pMCAO). Furthermore, we employed brain microvessel endothelial cells (BMECs) in an OGD model to mimic ischemic damage *in vitro*, and find out whether Shh regulates the ZO-1, occludin, claudin-5 expression, by regulating the expression of Ang-1.

## Materials and Methods

This study was carried out in strict accordance with the recommendations in the Guide for the Care and Use of Laboratory Animals of the National Institutes of Health. The protocol was approved by the Animal Care and Use Committee of Tongji Medical College of Huazhong University of Science and Technology (Permit Number: 2004-0007). All surgery was performed under chloral hydrate anesthesia, and all efforts were made to minimize suffering. Dead rats during operation were excluded from the experiments.

### Permanent Middle Cerebral Artery Occlusion

Male Sprague-Dawley (SD) rats, from the Center of Experimental Animals, Tongji Medical College of Huazhong University of Science and Technology (200–250 g), were housed in a controlled environment (12-h light/dark cycle, 22±2°C, 55±5% relative humidity) and allowed free access to food and tap water. Rats were anesthetized by intraperitoneal injection of 10% chloral hydrate (300 mg/kg). Rectal temperature was maintained at 37.3±0.5°C with a feedback-regulated heating pad during the procedures. Permanent middle cerebral artery occlusion (pMCAO) model was established in accordance with a previous report [20]. In brief, the right common carotid artery, right external carotid artery, and right internal carotid artery were isolated via a midline incision. The right external carotid artery was ligated with 6-0 nylon suture. Then, a poly-L-lysine–coated 4-0 nylon suture was inserted from the right internal carotid artery and advanced for about 20 mm to occlude the origin of right middle cerebral artery. Sham-operated rats underwent the same procedures but without thread insertion.

### Intracerebroventricular Injections

Immediately after pMCAO model establishment, the rats were mounted on a Digital stereotaxic apparatus (RWD Life Science, China) for drugs administration as previously described [21]. In brief, anesthetized rats were intracerebroventricularly injected 3 µL of vehicle (Phosphate Buffered Saline, PBS, Sigma, USA), 3 µL of Shh (1 mg/mL, Curis, Cambridge, USA), and 3 µL of Shh plus cyclopamine (10 mg/mL, LC laboratory, USA) [8]. Rats were placed on the stereotactic frame and injected at coordinate’s bregma −0.8 mm anteroposterior, ±1.5 mm mediolateral, and −4.5 mm dorsoventral [8]. Physiologic parameters remained in the normal range (body temperature (°C): 37.7±0.3; PaCO_2_ (mm Hg): 40.97±4.2; PaO_2_ (mm Hg): 131.73±73.97; pH: 7.087±0.08). Postoperatively, rats were left on the heating pad for maintaining the body temperature until they woke up. The drugs were injected on daily basis and the results were observed 1 d, 3 d, and 7 d after the injection. Animals were randomly divided into the following ten groups (n = 6 in each group): (1) sham-operated group, (2–4) ischemia (pMCAO 1 d, 3 d, and 7 d) and PBS groups, (5–7) ischemia (pMCAO 1 d, 3 d, and 7 d) and Shh groups, (8–10) ischemia (pMCAO 1 d, 3 d, and 7 d) and Shh plus cyclopamine groups. The animals that were neither operated nor medicated serve as controls. Each rat was given a discrete value of 0 (no apparent deficits), 1 (contralat forelimb flexion), 2 (decreased grip of the contralat forelimb while animal is pulled by tail), 3 (spontaneous movement in all directions; contralat circling only if animal is pulled by tail), 4 (spontaneous contralat circling) or 5 (death) to evaluate the neurological function [22]. During the experiments, rats exhibited no apparent deficits within the first evaluation or dead were excluded.

### Evaluation of Brain Edema

The water content was calculated as the weight difference between wet and dry samples. Rats (n = 6/group) were deeply anesthetized and sacrificed, by decapitation, at indicated time points (1 d, 3 d and 7 d) after pMCAO and medication. The brains were removed and immediately weighed to obtain the wet weight. Then they were dessicated at 100°C for 48 h and re-weighed to obtain the dry weight. The percentage of water in the brain was calculated as follows: (wet weight – dry weight)/wet weight×100%.

### Evaluation of Blood-Brain Barrier Permeability by Evans Blue Extravasation

The BBB permeability was quantitatively evaluated by using Evans blue (EB, Sigma, USA) as a marker of albumin extravasation, according to published protocols with some modification [23]. Briefly, before rats were sacrificed, 3 mL/kg of Evans blue (2%, Sigma, USA) in normal saline was injected into the tail vein of anesthetized rats, and then the vessels were perfused with PBS 4 h later. The brain was removed and divided into right and left hemispheres. Brain tissues were washed in PBS twice, blotted dry, weighed, homogenized in 1500 mL formamide, and incubated overnight in formamide at 55°C. The samples were centrifuged at 20000 g for 45 min. A supernatant was used to measure the absorbance of Evans blue at 620 nm with a spectrophotometer (Genesis 10 uv; Thermo Electron Corp., USA). Evans blue content was expressed as µg/g of brain tissue, which was calculated with the standard curve. To normalize the perfusion efficiency, the absorbance of the contralateral hemisphere was subtracted from the hemisphere ipsilateral to the pMCAO.

### TTC Staining

The ischemic model was confirmed by 2, 3, 5-triphenyltetrazolium chloride (TTC, Sigma, USA) staining. Briefly, after pMCAO and medication, the brain was quickly removed and sliced to five coronal sections (1 mm thickness) at +3.7, +1.0, −0.8, −3.3, and −5.3 mm from the bregma by using a Rat Brain Slicer Matrix (Zivic Instruments, USA). The brain slices were immersed in a 2% solution of TTC at 37°C for 30 min, and were then fixed in 10% phosphate-buffered formalin. Photographs were obtained and the ischemic penumbra was selected for analyzing the molecular of interest in the following experiments.

### Culture of Primary Rat Brain Microvascular Endothelial Cells (BMECs)

Brain microvascular endothelial cells were isolated from brains of SD rats (n = 4, 3–5 weeks of age), according to published protocols [24]. Briefly, rats were swabbed, killed and then their brains were harvested. The white matter, brain stem, surface vessels and leptomeninges were carefully removed. The isolated cerebral cortices were placed into PBS, minced into small pieces and then homogenized. The homogenates were centrifuged at 500 g for 5 min at 4°C. The pellet was re-suspended in 20% bovine serum antigen (BSA, Sigma, USA) and centrifuged at 1000 g for 20 min at 4°C. The microvessels in the lower layer were transferred to a new tube and washed once with PBS. The microvessel pellets were digested with 0.1% collagenase II/dispase (Sigma, USA) and 1000 U/ml DNase I (Sigma, USA) at 37°C for 1 h. After centrifugation at 500 g for 5 min at 4°C, the microvessel pellets were re-suspended in 10 mL M131 medium supplemented with Microvascular Growth Supplement, 100 U/ml penicillin and 100 U/ml streptomycin (Invitrogen, USA). The cell suspension was seeded onto a 75 cm^2^ flask (Corning, USA), and cells of 3–6 passage were used. BMECs were incubated at 37°C in humidified 5% CO_2_/95% air and identified by using immunofluorescence staining with goat anti-CD31 (1∶100, Santa Cruz Technology, USA).

### Culture of Primary Cortical Astrocytes

Primary cortical astrocytes from the brain tissue of 1-day-old Sprague–Dawley rats were cultured by using a technique we previously reported [6], [19]. The immunohistochemical staining showed that, on day 14, 95% of the cells were positive for glial fibrillary acidic protein (GFAP).

### Oxygen Glucose Deprivation and Drug Treatment

To mimic ischemic conditions, oxygen glucose deprivation (OGD) was used on the cells *in vitro*. For OGD studies, Ringer–HEPES solution (Gibco, USA) was first equilibrated with nitrogen and glucose was removed from the medium. On the day of the experiment, cells were exposed to OGD medium (low-glucose (0.2 g/L) equilibrated with nitrogen, Gibco, USA). PBS, Shh (3 µg/mL), cyclopamine (20 µM), and/or Ang-1-neutralizing antibody (l µg/mL), were added prior 30 min to the exposure to OGD. Cells in Gas Pack Pouch were incubated in an oven (Becton Dickinson, Franklin Lakes, NJ, USA) in 0% O_2_, 5%CO_2_, 95% N_2_ at 37°C for 4 h. Each experiment was performed in triplicate.

### ELISA Detection of Ang-1

BMECs (1.0×10^5^) or astrocytes (1.0×10^5^) were pre-treated with PBS, Shh and/or cyclopamine for 30 min and then subjected to OGD. Four hours after the exposure to OGD, supernatants (100 µL) were collected and centrifuged for 10 min. Commercial Ang-1 ELISA kit (R&D Systems, USA) was employed to determine the concentration of Ang-1 in each of the samples.

### Quantitative Real-time RT-PCR

Total RNA of cerebral cortices obtained from ischemic penumbra or cultured cells treated with PBS, Shh, Ang-1-neutralizing antibody and/or OGD was extracted and reversely transcribed into cDNA. The cDNA was then used as a template for quantitative real-time RT-PCR, which was performed by using the Super-Script III First-Strand Synthesis System (Invitrogen, USA) by following the manufacturer’s instructions. The mRNA levels of ZO-1, occludin, claudin-5 were determined by quantitative real-time RT-PCR by using the SYBR Green PCR Master Mix (Invitrogen, USA). The specific primers are listed in Table 1. GAPDH was used as internal control for the normalization of gene expression.

**Table 1:** Sequences of primers.

Gene name	Forward primer (5′-3′)	Reverse primer (5′-3′)	Size
ZO-1	CACGATGCTCAGAGACGAAGG	TTCTACATATGGAAGTTGGGGATC	156
Occludin	GCAAAGTGAATGGCAAGAGATC	CGTGTAGTCGGTTTCATAGTGGTC	227
Claudin-5	TTCGCCAACATCGTAGTCCGCTC	TCTTCTTGTCGTAATCGCCG	110
Ang-1	TGGTGAATATTGGCTTGGG	CAGTTGTCGTTATCAGCGTCCT	252

### Western Blotting

After pMCAO establishment and medication (1 d, 3 d, and 7 d), the tissue samples from the ischemic penumbra were taken, put into the lysis buffer containing protease inhibitor, homogenized, and centrifuged. Protein (30 µg) was subjected to 12% SDS-polyacrylamide gels and electrophoretically transferred to nitrocellulose membranes. Membranes were incubated with specific primary antibodies: polyclonal goat anti-ZO-1 (1∶500, Santa Cruz Technology, USA), rabbit anti-occludin (1∶200, Abcam, USA), rabbit anti-Claudin-5 (1∶200, Santa Cruz Technology, USA), and goat anti-Ang-1 (1∶200, Abcam, USA), and then incubated with horseradish peroxidase-conjugated secondary antibody (ICN Pharmaceuticals, USA). Proteins were visualized by using a Super Signal West Pico chemiluminescence kit (Thermo Scientific, USA). β-actin (1∶500, Santa Cruz Technology, USA) served as internal control. The protein bands were scanned by using Chemi Imager 5500 V2.03 software and integrated density values (IDV) were calculated by using Image J software package (NIH Shareware), with β-actin serving as internal control.

### Statistical Analysis

All values are expressed as means ± standard deviation (SD). Statistical analyses were performed by using one-way ANOVA followed by *post hoc* comparison by using Bonferroni/Dunn test. *P*<0.05 was considered to be statistically significant. Statistical analyses were performed by employing the Statistical Package for the Social Sciences (SPSS 13.0, USA) software.

## Results

### Shh Treatment Ameliorates the Brain Edema

**Figure 1:**
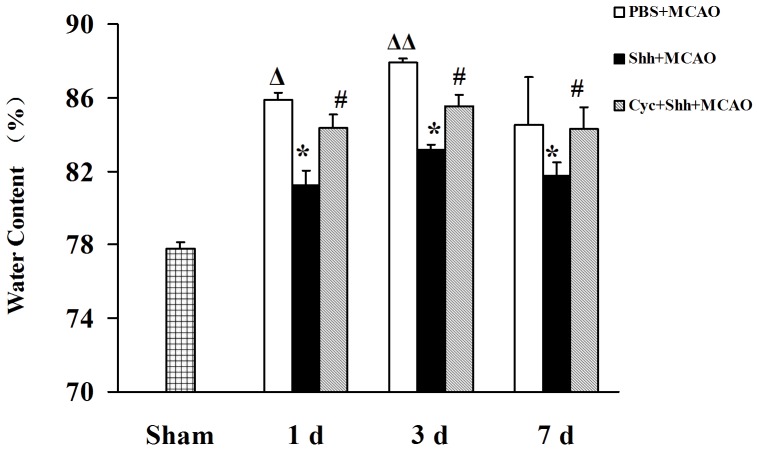
Shh Treatment Ameliorates the Brain Edema. After establishment of pMCAO, rats were intracerebroventricularly injected 3 µL of PBS, 3 µg of Shh, and Shh (3 µg) plus cyclopamine (Cyc, 30 µg). The brain edema was detected by water content measurement at indicated time points (1 d, 3 d, and 7 d). The bar graph shows that Shh decreased the brain edema in pMCAO and the effect was reversed by the Shh-specific antagonist Cyc. In Sham group, rats received 3 µL of PBS without undergoing pMCAO operation. In PBS+pMCAO group, rats received 3 µL of PBS after pMCAO. In Shh+pMCAO group, rats were given 3 µg of Shh after pMCAO. In Cyc+Shh+pMCAO group, rats were received 3 µg of Shh and 30µg of Cyc after pMCAO. ^Δ^ *P*<0.01 vs. the Sham group, *P<0.01 vs. the PBS group at the same time point, ^#^ *P*<0.01 vs. the Shh group at the same time point, ^ΔΔ^ *P*<0.01 vs. the PBS (1 d) group. Each experiment was repeated three times (*n* = 6, each group).

Brain edema was measured by relative water content in all groups. Rat treated with Shh showed significantly reduced water content 1 d (81.24±0.08%, *P<0.01 *vs.* the PBS group), 3 d (83.15±0.28%, *P<0.01 *vs.* the PBS group) and 7 d (81.74±0.73%,*P<0.01 *vs.* the PBS group) after the treatment, as compared with rat treated with PBS under the same condition (85.89±0.41%; 87.91±0.25%; 84.49±2.61%; Fig. 1; ^Δ^ *P*<0.01 *vs.* the Sham group, ^ΔΔ^ *P*<0.01 *vs.* the PBS (1 d) group). Moreover, cyclopamine could reverse the effects of Shh on brain edema (84.34±0.73%; 85.54±0.61%; 84.32±1.14%, ^#^ *P*<0.01 *vs.* the Shh group). Cyclopamine, an alkaloid that binds Smo and thereby inhibits Gli-1 activation, has been shown to effectively and specifically block Shh signaling pathway [25].

### Shh Treatment Preserves the Permeability of BBB

**Figure 2:**
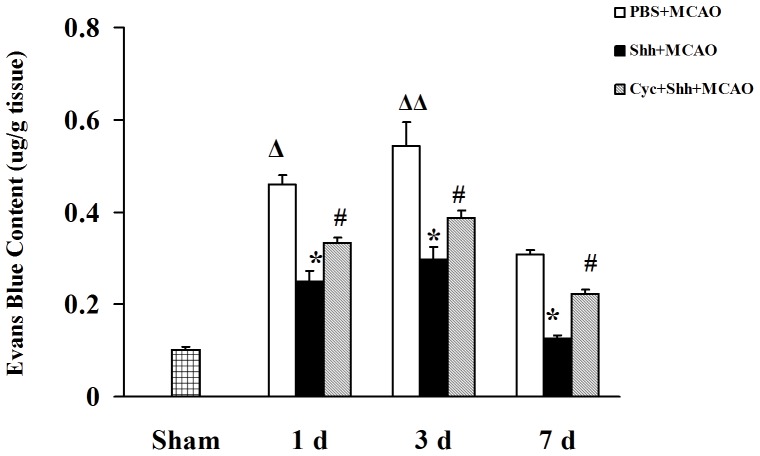
Shh Treatment Preserves BBB permeability. After establishment of pMCAO, rats were intracerebroventricularly injected 3 µL of PBS, 3 µg of Shh, and Shh (3 µg) plus cyclopamine (Cyc, 30 µg). The BBB permeability was detected by Evans Blue extravasation at indicated time points (1 d, 3 d, and 7 d). Bar graph shows that Shh decreased the BBB leakage in pMCAO and the effect was reversed by the specific Shh antagonist Cyc. Grouping was the same as above. ^Δ^ *P*<0.01 vs. the Sham group, *P<0.01 vs. the PBS group at the same time point, ^#^ *P*<0.01 vs. the Shh group at the same time point, ^ΔΔ^ *P*<0.01 vs. the PBS (1 d) group. Each experiment was repeated three times (n = 6, each group).

We then determined the effect of Shh on the permeability of BBB by examining the relative Evans blue contents in the ischemic penumbra of brain cortex. The results showed that the Evans blue contents at 1 d were increased in pMCAO groups (0.46±0.02 µg/g), as compared with the ipsilateral cortex of the sham group (0.10±0.01µg/g, ^Δ^ *P<0.01 vs.* the Sham group). Evans blue extravasation revealed that rats treated with Shh had a significantly lower contents of Evans blue in the ischemic brain cortex 1 d (0.25±0.02 µg/g, *P<0.01 *vs.* the PBS group), 3 d (0.30±0.03 µg/g, *P<0.01 *vs.* the PBS group), and 7 d (0.13±0.01 µg/g, *P<0.01 *vs.* the PBS group) after the treatment, as compared with rats treated with PBS under the same time conditions (0.46±0.02, 0.54±0.05, 0.31±0.01 µg/g, ^Δ^ *P*<0.01 *vs.* the Sham group; ^ΔΔ^ *P*<0.01 *vs.* the PBS (1 d) group), while cyclopamine could inhibit the effects of Shh (0.33±0.01, 0.39±0.02, 0.22±0.01 µg/g, ^#^ *P*<0.01 *vs.* the Shh group, Fig. 2).

### Shh Treatment Upregulates the mRNA and Protein Expressions of ZO-1 and Occludin *in vivo and in vitro*

**Figure 3:**
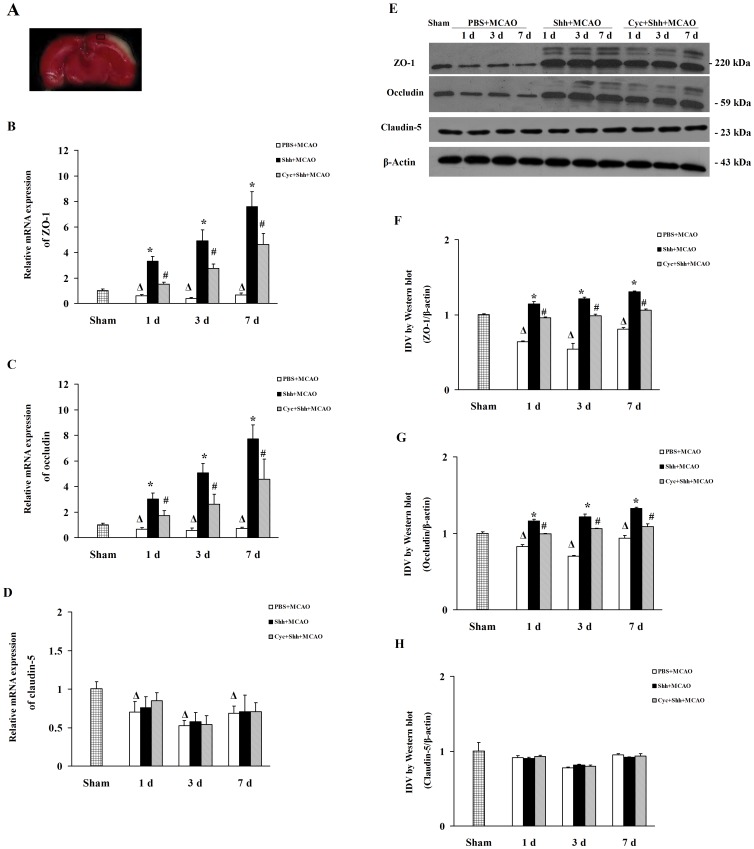
Shh Treatment Upregulates the Expression of ZO-1 and Occludin In the Brain Tissue of Ischemic Rats. After establishment of pMCAO, rats were intracerebroventricularly injected 3 µL of PBS, 3 µg of Shh, and Shh (3 µg) plus cyclopamine (Cyc, 30 µg). The brain slices were staining by using a 2% TTC solution, and we selected the ischemic penumbra for the ensuing experiments. The expression of ZO-1, occludin and claudin-5 from ischemic penumbra was detected by Western blotting and real-time RT-PCR at indicated time points (1 d, 3 d, and 7 d). (A) Representative photographs of TTC stained brain slice. Brain samples were selected from ischemic penumbra. (B-D) The mRNA levels of ZO-1 (B), occludin (C) and claudin-5 (D). (E) Representative photographs of Western Blotting bands. (F-G) The IDV of ZO-1, occludin, and claudin-5. Grouping was the same as above. ^Δ^ *P*<0.01 vs. the Sham group, *P<0.01 vs. the PBS group at the same time points, ^#^ *P*<0.01 vs. the Shh group at the same time points. Each experiment repeated three times (n = 6, each group).

The expressions of ZO-1, occludin and claudin-5 were detected with real-time RT-PCR and western blotting, respectively. Fig. 3B–D show that the mRNA expression levels of ZO-1 and occludin are decreased significantly in pMCAO plus PBS group 1 d (0.61±0.09; 0.68±0.12, ^Δ^ *P*<0.01 *vs.* the Sham group), 3 d (0.39±0.08; 0.57±0.19, ^Δ^ *P*<0.01 *vs.* the Sham group), and 7 d (0.66±0.16; 0.71±0.12, ^Δ^ *P*<0.01 *vs.* Sham group) after the treatment as compared with the sham group (1.01±0.13). Administration of Shh significantly increased the mRNA expression level of ZO-1 (3.32±0.38, 4.92±0.84, 7.58±1.68, *P<0.01 *vs.* the PBS group) and occludin (3.03±0.46, 5.08±0.71, 7.72±1.09, *P<0.01 *vs.* the PBS group) after the treatment as compared with the pMCAO plus PBS group, and the effects could be reversed by cyclopamine (ZO-1∶1.50±0.68, 2.74±0.34, 4.63±0.85, ^#^ *P*<0.01 *vs.* the Shh group; occludin: 1.75±0.38, 2.60±0.81, 4.57±1.58, ^#^ *P*<0.01 *vs.* the Shh group).

Fig. 3E shows the representative immunoblots of ZO-1, occludin and claudin-5 during pMCAO. Fig. 3F–3H shows the IDV changes of ZO-1, occludin, and claudin-5 protein during pMCAO. The IDV changes of ZO-1 and occludin are significantly decreased in pMCAO plus PBS group 1 d (0.64±0.01; 0.83±0.03, ^Δ^ *P*<0.01 *vs.* the Sham group), 3 d (0.54±0.08; 0.70±0.01, ^Δ^ *P*<0.01 *vs.* the Sham group), and 7 d (0.81±0.02; 0.94±0.03, ^Δ^ *P*<0.01 *vs.* Sham group) after the treatment as compared with the sham group (1.00±0.01; 1.00±0.02). Administration of Shh significantly increased the protein expression level of ZO-1 (1.15±0.03, 1.22±0.02, 1.31±0.01, *P<0.01 *vs.* the PBS group) and occludin (1.16±0.03, 1.21±0.04, 1.32±0.01, *P<0.01 *vs.* the PBS group) as compared with the pMCAO plus PBS group, and the effects could be reversed by cyclopamine (ZO-1∶0.96±0.01, 0.98±0.02, 1.06±0.01, ^#^ *P*<0.01 *vs.* the Shh group; occludin: 0.99±0.01, 1.06±0.01, 1.08±0.04, ^#^ *P*<0.01 *vs.* the Shh group). These results are consistent with the RT-PCR results. Moreover, Shh exerts no effect on the mRNA and protein expression of claudin-5 under ischemia.

**Figure 4:**
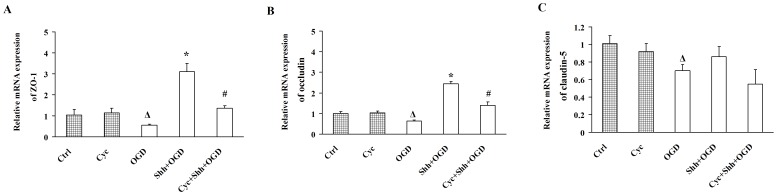
Shh Treatment Upregulates the Expression of ZO-1 and Occludin in BMECs under OGD. BMECs were pre-treated with PBS, Shh and/or cyclopamine (Cyc) for 30 min, and then subjected to the OGD for 4 h. (A-C) The mRNA levels of ZO-1 (A), occludin (B) and claudin-5 (C) were determined by real-time RT-PCR. Ctrl: cells were pre-treated with PBS and then subjected to normal oxygen condition. Cyc: cells were pre-treated with Cyc and then subjected to normal oxygen condition. OGD: cells were pre-treated with PBS and then subjected to OGD. Shh+OGD: cells were pre-treated with Shh and then subjected to OGD. Cyc+Shh+OGD: cells were pre-treated with Shh plus Cyc and then subjected to OGD. ^Δ^ *P*<0.01 vs. the Ctrl group, *P<0.01 vs. the OGD group, ^#^ *P*<0.01 vs. the Shh group. Each experiment was repeated three times (n = 6, each group).

We then examined the effects of Shh on ZO-1, occludin and claudin-5 expression of BMECs under OGD, and found that, under OGD for 4 h, pre-treated with Shh significantly increased the mRNA expression of ZO-1 (3.10±0.40, *P<0.01 *vs.* the OGD group) and occludin (2.45±0.12, *P<0.01 *vs.* the OGD group) as compared with OGD group (0.56±0.05; 0.64±0.05; ^Δ^ *P*<0.01 *vs.* the Control group), and the effects could be reversed with cyclopamine (1.36±0.12; 1.40±0.16,^ #^ *P*<0.01 *vs.* the Shh group, Fig. 4A–4C). Shh exerted no effect on the expression of claudin-5.

### Shh Treatment Upregulates the mRNA and Protein Expressions of Ang-1 *In Vivo* and *In Vitro*

**Figure 5:**
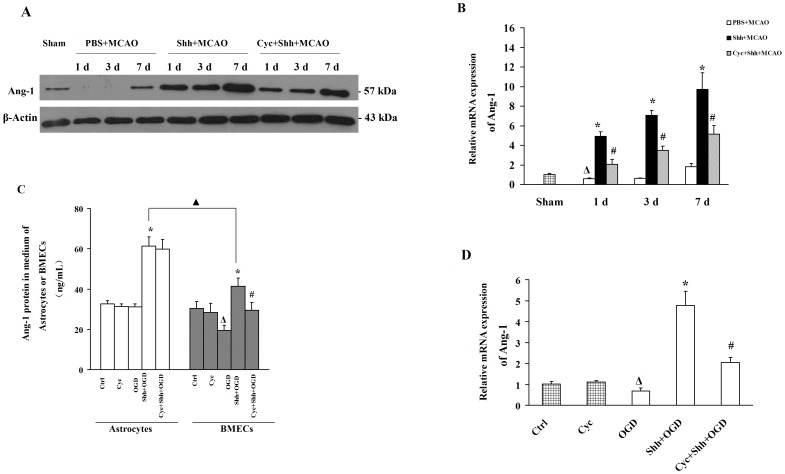
Shh Treatment Upregulates the Expression of Ang-1 Under Ischemic Condition *in vivo* and *in vitro*. (A–B) After establishment of pMCAO, rats were intracerebroventricularly injected 3 µL of PBS, 3 µg of Shh, and Shh (3 µg) plus cyclopamine (Cyc, 30 µg). The expression of Ang-1 was detected by Western blotting and real-time RT-PCR at indicated time points (1 d, 3 d, and 7 d). (A) Representative photographs of Western Blotting bands. (B) The mRNA levels of Ang-1. Grouping was the same as above. (C) BMECs or astrocytes were pre-treated with PBS, Shh and/or Cyc for 30 min, and then subjected to the OGD for 4 h. The concentration of Ang-1 protein in the supernatal of cells in different groups was determined by ELISA. (D) BMECs were pre-treated with PBS, Shh and/or Cyc for 30 min, and then subjected to the OGD for 4 h. The mRNA levels of Ang-1 were determined by real-time RT-PCR. Grouping was the same as above. ^Δ^ *P*<0.01 vs. the Ctrl group, *P<0.01 vs. the OGD group, ^#^ *P*<0.01 vs. the Shh group. ^▴^ *P*<0.01 vs. the BMECs group. Each experiment was repeated three times (n = 6, each group).

Real-time RT-PCR and western blotting were used to examine the effects of Shh on the expression of Ang-1 in different groups. Fig. 5A–5B show that the mRNA and protein expressions of Ang-1 are increased 7 d after pMCAO plus PBS. Shh increased the mRNA level of Ang-1 in a time-dependent manner. Compared with the PBS-treated group (1 d, 3 d and 7 d, 0.59±0.10, 0.63±0.06, 1.82±0.31, respectively), the relative expression of Ang-1 in the Shh-treated group was significantly increased (4.91±0.49, 7.06±0.50, 9.71±1.17, respectively, *P<0.01 *vs.* the PBS group; Fig. 5B). Shh also increased the protein expression of Ang-1 as compared with the PBS group (Fig. 5A). Cyclopamine could reverse the effects of Shh on the expression of Ang-1 in ischemic brain tissue.

To specifically identify the source of Ang-1 in response to Shh, we detected the content of secreted Ang-1 protein in medium of astrocytes or BMECs under OGD and medication. ELISA showed that, the Ang-1 protein secreted into the medium of astrocytes was significantly higher in Shh-treated group (61.30±4.42 ng/mL, *P<0.01 *vs.* the OGD group) than in OGD group (31.20±1.31 ng/mL), Cyclopamine could not reverse the effects of Shh (59.95±4.74 ng/mL, Fig. 5C). In the BMECs, the Ang-1 protein secreted into the medium was also significantly higher in Shh-treated group (41.28±4.16 ng/mL, *P<0.01 *vs.* the OGD group) than in OGD group (19.43±2.62 ng/mL). Cyclopamine could reverse the effects of Shh (29.45±3.85 ng/mL, *P<0.01 *vs.* the Shh group, Fig. 5C). Moreover, the Ang-1 protein concentration in culture medium of Shh-treated astrocytes was significantly higher (61.30±4.42 ng/mL,^ ▴^ *P*<0.01 *vs.* the BMECs group) than in BMECs culture medium (41.28±4.16 ng/mL, Fig. 5C).

Real-time RT-PCR revealed that the mRNA expression of Ang-1 in BMECs was significantly higher in Shh-treated group (4.75±0.68, *P<0.01 *vs.* the OGD group; Fig. 5D) as compared with the OGD group (0.67±0.15). The effects of Shh could be reversed by cyclopamine (2.04±0.25, ^#^ *P*<0.01 *vs.* the Shh group).

### Treatment with Ang-1-neutralizing Antibody Suppresses Shh-up-regulated ZO-1 and Occludin in BMECs under OGD

**Figure 6:**
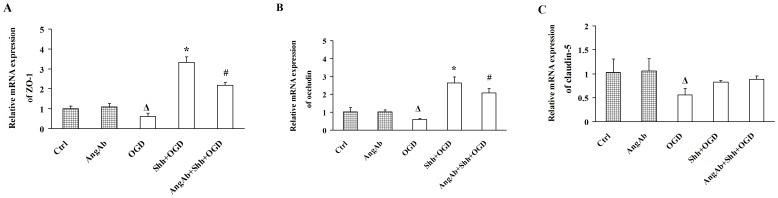
Treatment with Ang-1-neutralizing Antibody Suppresses Shh-up-regulated ZO-1 and Occludin in BMECs under OGD. BMECs were pre-treated with Ang-1-neutralizing antibody (AngAb), and/or PBS, Shh for 30 min, and then subjected to the OGD for 4 h. (A-C) The mRNA levels of ZO-1 (A), occludin (B) and claudin-5 (C) were determined by real-time RT-PCR. Ctrl: cells were pre-treated with PBS and then subjected to normal oxygen condition. AngAb: cells were pre-treated with AngAb and then subjected to normal oxygen condition. OGD: cells were pre-treated with PBS and then subjected to OGD. Shh+OGD: cells were pre-treated with Shh and then subjected to OGD. AngAb+Shh+OGD: cells were pre-treated with Shh plus AngAb and then subjected to OGD. ^Δ^ *P*<0.01 vs. the Ctrl group, *P<0.01 vs. the OGD group, ^#^ *P*<0.01 vs. the Shh group. Each experiment was repeated three times (n = 6, each group).

After pre-treatment with Ang-1-neutralizing antibody, the increased mRNA level of ZO-1 and occludin of BMECs in Shh-treated group (3.33±0.27; 2.64±0.33) was significantly decreased under OGD (0.61±0.17; 0.60±0.05, *P<0.01 *vs.* the Shh group; Fig. 6A and 6B). Pre-treated with Ang-1-neutralizing antibody exerted no effects on the expression of claudin-5 (Fig. 6C).

## Discussion

The elements composing the blood–brain barrier (BBB) are endothelial cells, pericytes, and the end-feet of astrocytes. Among them, because of intact tight junction between brain capillary cells are critical for normal brain barrier function, the endothelial cell barrier line is the most critical for preventing toxic substances from entering the brain [26]. Alteration of the tight junction protein results in brain edema after focal cerebral ischemia. In this study, we demonstrated that administration of Shh acts on tight junction proteins of BMECs to preserve permeability of the BBB under ischemic insults in vitro and in vivo, a process that is Smo-dependent. We further demonstrate that Shh regulates activation of tight junction proteins of BMECs by increasing the expression of Ang-1.

Sonic hedgehog (Shh), as a secreted protein of Hh signaling pathway, plays an important role in vascular proliferation, differentiation and maturation [27], [28]. Recent studies showed that the component of Shh signaling pathway are increased after ischemia in some tissues such as myocardium and brain [29], [7]. Furthermore, inhibition of Shh signaling pathway aggregates the level of brain edema in ischemic stroke [8]. In this study, we found that the brain edema was eliminated in Shh treated animals, and the effects could be reversed by cyclopamine. Consistent with the published report, the difference of water content is statistically significant, although the actual difference is less than 5%. Cyclopamine, as a Smo antagonist, specifically inhibits the Shh signaling pathway [25].

Tight junctions’ proteins are important for the BBB integrity and were found regulated by Shh [4]. Furthermore, Shh null mice exhibit developmentally arrested submandibular gland epithelium, whereas treatment with Shh enhances the formation of epithelial lumens and the distribution of ZO-1, claudin-3 and occludin [30]. ZO-1 is a well characterized tight junction protein and can accurately reflect the pathological changes of BBB, making it a valuable marker of endothelial barrier [31]. It has been shown that absence of ZO-1 results in vascular leakage and the aggravated edema is likely due to a decreased level of the tight junction protein ZO-1 [32]. Occludin is also highly expressed in barrier endothelia and acts as a key TJ protein whose level dictates tissue barrier properties [33]. Increased expression of occludin correlates with improved barrier function through elevating trans-epithellal electric resistance (TEER) in several cell lines [34]. In this study, we found that Shh can increase the expression of ZO-1 and occludin in BMECs under OGD. Our previous study found that, BMECs is likely one of the targeted cells by Shh [35]. The neurons and astrocytes are also the targeted cells of Shh [5], [6]. Moreover, the presence of ZO-1 and occludin is the characteristic feature of BMECs [36], and recent work found that, the expression of occludin and ZO-1 is decreased by blocking Shh signaling in vitro and using Shh knock-out (−/−) embryonic mice [4]. Taken together, our data suggest that Shh treatment upregulates the expressions of ZO-1 and occludin in BMECs.

The activation of the Shh signaling pathway involves activation of the downstream target gene including Ang-1 [12], [18]. Ang-1 is a strong anti-permeability factor that could reduce vascular leakage. In this study, we observed that the expression of Ang-1 is significantly decreased during the first 3 days after injury, when cerebral edema is most serious. Furthermore, the BBB leakage is significantly reduced, as shown by extravasation of Evans blue. This dynamic change in Ang-1 expression may explain the progression of cerebral edema after ischemia and suggest that Ang-1 acts at a later stage of vascular stabilization and maturation.

Administration of Shh could increase the expression of Ang-1 at the early stage in ischemic penumbra. In line with our previously study [19], we found that Shh significantly upregulated Ang-1 in astrocytes, and this effect could not be reversed by cyclopamine, suggesting that Shh induce Ang-1 in astrocytes not through Gli-1. NR2F2 may be involved in it. Additionally, the primary brain microvascular endothelial cells used in this study were found to be more sensitive to exogenous Shh, and Shh could increase Ang-1 expression in these cells [4], [37]. We also found that Shh upregulated Ang-1 in BMECs, and this effect could be reversed with cyclopamine, suggesting that Shh induces Ang-1 in BMECs via Gli-1. Importantly, we also found that the content of secreted Ang-1 of BMECs was lower than that of astrocytes, suggesting the cells with elevated Ang-1 in response to Shh were mainly astrocytes. Ang-1 is involved in vascular maturation and quiescence, and could cause a time- and dose-dependent decrease in endothelial permeability by up-regulating tight junction-associated proteins [17], [38]. A number of studies showed that Ang-1 could regulate the expression of ZO-1 and occludin [14], [39]. Furthermore, a recent study showed that Ang-1 may decrease the permeability of BBB in brain ischemia by up-regulating the expression of ZO-1 and occludin [40]. Our study demonstrated that inhibiting Ang-1 with Ang-1-neutralizing antibody reversed the effects of Shh on the up-regulated expression of ZO-1 and occludin in BMECs under OGD. These results suggest that Ang-1 derived from astrocytes may be implicated in the maintenance of BBB integrity regulated by Shh.

However, little is known about the mechanisms of Ang-1 regulating the endothelial barrier. A previous research found that Ang-1 is able to block the structural rearrangements and hyperpermeability through PI3K, which activate Rac1, phosphorylate p190 RhoGAP, and inhibit RhoA activity [41]. Our previous study found RhoA/ROCK pathway is activated by astrocytes to promote the tube Formation and Migration of BMECs [36]. Further studies are needed to investigate whether co-culturing BMECs and astrocytes play a role in BBB permeability and Ang-1 involved in it.

To sum up, the present study demonstrated that, under ischemic insults, Shh triggers Ang-1 production principally in astrocytes, and the secreted Ang-1 acts on BMECs, thereby upregulating ZO-1 and occludin to repair the tight junction and ameliorate the brain edema and BBB leakage.

## Supporting Information

Reply letter 2 (DOC)

Reply letter (DOC)
