# Supplementary material for: Recombinant Human Sonic Hedgehog Protein Regulates the Expression of ZO-1 and Occludin by Activating Angiopoietin-1 in Stroke Damage
Source: PLoS One. 2013 Jul 23;8(7):e68891. doi: 10.1371/journal.pone.0068891 (PMC3720889; doi:10.1371/journal.pone.0068891)
Supplement: Reply letter 2 — (DOC) [file pone.0068891.s001.doc]

Dear editor:

We thank you for your attention.

1. Chloral hydrate is considered an unacceptable anesthetic agent for small animals. Please provide justification for choosing this method of anesthesia over other available methods.

Answer: The surgery of pMCAO model and the anesthetic method was followed by Belayev et al., 1996 and Shen et al., 2011. The anesthetic method was very convenient and no animals died because of the anesthesia. However, after your guidance, we could update our methods in the following experiments. Thank you very much.

2. Please describe all efforts to alleviate suffering, outside of what is already presented in the manuscript. How often were animals monitored and given deficit scores? What was the method of euthanasia when one was used?

Answer: The animals were monitored and given deficit scores every day. The Chloral hydrate was used for euthanasia before the operation and experiments. During and after operation, animals were always put in the comfortable environments, and after experiments, the dead body were given humane treatment following the protocols approved by the Animal Care and Use Committee of Tongji Medical College of Huazhong University of Science and Technology

3. How many animals reached a deficit score of 5 post-operatively? Were these animals allowed to die, or were they euthanized? If they were not euthanized, please provide justification for this approach.

Answer: 60 of animals reached the deficit score post-operatively, and they euthanized and sacrificed before the experiments.

4. Were all animals that died during surgery under deep anesthesia before death? If not, please provide justification for this approach.

Answer: Before experiments for harvesting specimen, the animals were euthanized deeply before sacrificed. During the surgery of pMCAO and drug treatments, the animals were just euthanized, and they could wake up and be alive in the comfortable enviroments.

I look forward to hearing from you.

Sincerely,

Bo Hu
