# Supplementary material for: Recombinant Human Sonic Hedgehog Protein Regulates the Expression of ZO-1 and Occludin by Activating Angiopoietin-1 in Stroke Damage
Source: PLoS One. 2013 Jul 23;8(7):e68891. doi: 10.1371/journal.pone.0068891 (PMC3720889; doi:10.1371/journal.pone.0068891)
Supplement: Reply letter — (DOC) [file pone.0068891.s002.doc]

Dear editor:

We thank you for your attention.

1.Other than for tissue harvesting, did any animals die as a result of intracerebroventricular injection?

Answer: yes, some animals died because of the intracerebroventricular injection, and these animals were excluded from our groups. The number of rats dead during or after operation is 20.

2. If animals died, did they die on their own or were they euthanized beforehand?

Answer: The animals were dead on their own.

3. If animals were euthanized, what criteria were used to determine that animals should be euthanized and what was the method of sacrifice?

Answer: The animals were dead on their own.

I look forward to hearing from you.

Sincerely,

Bo Hu
